# Empirical assessment of the impact of sample number and read depth on RNA-Seq analysis workflow performance

**DOI:** 10.1186/s12859-018-2445-2

**Published:** 2018-11-14

**Authors:** Alyssa Baccarella, Claire R. Williams, Jay Z. Parrish, Charles C. Kim

**Affiliations:** 1 Division of Experimental Medicine, Department of Medicine, University of California, San Francisco, California 94143 USA; 2 Department of Biology, University of Washington, Seattle, WA 98195 USA; 3 Molecular and Cellular Biology Program, University of Washington, Seattle, WA 98195 USA; 4 Present address: Verily, South San Francisco, California 94080 USA

**Keywords:** Monocytes, RNA-sequencing, Gene expression analysis, Read depth, Sample number

## Abstract

**Background:**

RNA-Sequencing analysis methods are rapidly evolving, and the tool choice for each step of one common workflow, differential expression analysis, which includes read alignment, expression modeling, and differentially expressed gene identification, has a dramatic impact on performance characteristics. Although a number of workflows are emerging as high performers that are robust to diverse input types, the relative performance characteristics of these workflows when either read depth or sample number is limited–a common occurrence in real-world practice–remain unexplored.

**Results:**

Here, we evaluate the impact of varying read depth and sample number on the performance of differential gene expression identification workflows, as measured by precision, or the fraction of genes correctly identified as differentially expressed, and by recall, or the fraction of differentially expressed genes identified. We focus our analysis on 30 high-performing workflows, systematically varying the read depth and number of biological replicates of patient monocyte samples provided as input. We find that, in general for most workflows, read depth has little effect on workflow performance when held above two million reads per sample, with reduced workflow performance below this threshold. The greatest impact of decreased sample number is seen below seven samples per group, when more heterogeneity in workflow performance is observed. The choice of differential expression identification tool, in particular, has a large impact on the response to limited inputs.

**Conclusions:**

Among the tested workflows, the recall/precision balance remains relatively stable at a range of read depths and sample numbers, although some workflows are more sensitive to input restriction. At ranges typically recommended for biological studies, performance is more greatly impacted by the number of biological replicates than by read depth. Caution should be used when selecting analysis workflows and interpreting results from low sample number experiments, as all workflows exhibit poorer performance at lower sample numbers near typically reported values, with variable impact on recall versus precision. These analyses highlight the performance characteristics of common differential gene expression workflows at varying read depths and sample numbers, and provide empirical guidance in experimental and analytical design.

**Electronic supplementary material:**

The online version of this article (10.1186/s12859-018-2445-2) contains supplementary material, which is available to authorized users.

## Background

RNA sequencing (RNA-Seq), despite being the choice technique for transcriptome-wide identification of differentially expressed genes, is still rapidly evolving. A number of tools are available for each major processing step in data analysis: read alignment, expression modeling, and differential gene identification [1], but their performance in concert is only beginning to be understood [2–6]. Additionally, most existing evidence concerning their performance has been built on samples from simulated or highly controlled datasets, which lack the variability inherent in many experimental—particularly clinically-derived—datasets. We recently performed a broad comparison of RNA-Seq differential expression analysis workflows, applied to human clinical samples of highly purified monocyte subsets, using previously published microarray and BeadChip data as our reference gene sets. We found that a number of workflows performed poorly, but that the majority of workflows perform similarly well, with differences in their calibration with respect to having higher recall or precision [7].

Although the cost of RNA-Seq experiments has been steadily decreasing as technology improves, it continues to be an important consideration when designing RNA-Seq experiments. In particular, a large component of that cost is derived from the sample preparation and sequencing, where a tradeoff between read depth and sample number (e.g. the number of biological replicates per condition) must be taken into consideration [8]. Currently there is no consensus on adequate sequencing depth for differential gene expression studies, and several studies on multiple organisms and sample types, varying in their heterogeneity, have suggested a wide range of read depths as optimal. At the low end, increasing read depths beyond 10 million reads was found to have minimal effect on the power to identify the effects of hormone treatment on a breast cancer cell line [8]; at the high end, increasing read depth up to 200 million reads, the highest depth available in the study, led to an increase, albeit small, in the detection of differentially expressed genes when comparing three male and three female human monocyte samples [9]. In simulations of three murine and four human data sets, including cell line, viral infection, disease, and population studies, a minimal power gain was seen above 20 million reads [10] or 30 million reads [11]. In studies employing one versus one sample comparisons, 200 million reads were suggested to identify the full range of differentially expressed transcripts between the MAQC dataset and a colorectal cancer line, and between human liver and kidney cells [12]; whereas 300 million reads was the depth identified as necessary to identify 80% of differentially expressed genes in human subcutaneous fat, pre and post induction of an inflammatory response [13].

Several studies have examined the interplay of sample number with read depth, with general consensus that increasing biological replicates increases power or gene recall more drastically than increasing read depth [8, 10, 14, 15]. However, similar to read depth, recommendations for sample number vary. At the lower end of recommendations, three to four samples were determined to be sufficient for differential gene identification in a mouse neurosphere study, based on the relatively small incremental improvements in the AUC at these sample numbers [15], and three samples per group were necessary to detect the majority of differences tied to genotype, sex, and environment in a *Drosophila melanogaster* study, although additional replicates did increase power [16]. Ching et al. recommended a minimum of five samples per group, based on a variety of RNA-Seq datasets, but both they and others noted that much higher sample numbers are necessary to provide adequate power in samples with high gene dispersion, such as in a population comparison of Caucasian and Nigerian derived cells [10, 15]. Similarly, in a *S. cerevisiae* study, a minimum of six replicates was recommended for differential expression studies, based on identification of true positive and false positive genes, although twelve replicates were necessary to identify the majority of differentially expressed genes [17]. It is likely that the wide range of organisms studied, including the genomic complexity, level of genetic heterogeneity within sample groups, the magnitude of differences in conditions, and the target fraction of differences to be identified, impacts these disparate results across read depth and sample number. Beyond attempting to reach a generalization about input design, it is even harder to extrapolate these findings to clinically-derived samples. Several studies have proposed methods for sample size calculation [10, 18–23]; however, their performance on real world data has shown widely variable results in sample size estimation, without clear indication that one outperforms the others [24]. As such, there remains a need for empirical studies of sample size effect, with a particular need for studies on experimental conditions more similar to study designs increasingly found in the literature.

Due to the paucity of real-world RNA-Seq samples for which reference datasets are available for comparison, studies of RNA-Seq tools have frequently been limited to using up-sampled RNA-Seq data as a metric of truth [8, 10, 13] or relied on highly controlled datasets generated in silico [10, 11, 15]. Additionally, many of the datasets frequently used for evaluation of RNA-Seq performance, such as the MAQC dataset [25] and the human kidney and liver dataset [26], do not allow for the study of effects of biological replicates, and exhibit extreme differences in gene expression that are not representative of typical study designs, where test groups are often much more closely related. Furthermore, many of the aforementioned studies only use a single workflow for differential gene identification, with none examining the interplay of tool choice at the three levels (read alignment, expression modeling, and differential expression) with read depth and sample number. Altogether, the limitations of these studies reflect the challenge of grappling with the multi-factorial parameters that can dramatically influence the performance of RNA-Seq analysis, and highlight the need for further assessments.

Here, we examined the effects of read depth and sample number on the performance of several differential expression analysis workflows, which have previously been identified as good performers when applied to input datasets with high read depth and sample number [7]. Performance was assessed using real-world, clinical samples of highly purified monocytes, with the use of four previously published microarray and BeadChip studies as a reference for biological truth [27–30]. The results of this study provide empirically-derived guidance to inform the design of RNA-Seq experiments, including the choice of RNA-Seq analysis workflow.

## Methods

### RNA-Seq samples

The RNA-Seq datasets used in this study were previously published [7] and are available from the NCBI Sequence Read Archive (SRA) under accession number SRP082682.

### Read subsampling, alignment, expression modeling, and differential expression identification

FASTQ files were randomly subsampled without replacement to create samples of depths 3 × 10^4^, 5 × 10^4^, 1 × 10^5^, 3 × 10^5^, 5 × 10^5^, 1 × 10^6^, 2 × 10^6^, 5 × 10^6^, 1 × 10^7^, and 2 × 10^7^ reads using the R package ShortRead [31], as allowed for by the original read depth of the sequenced sample (Additional file 1). Each subset of reads was aligned to release GRCh38 of the human genome (Gencode Release 26) with HISAT2, Kallisto, Salmon, and STAR [32–35]. Gene expression was modeled with Kallisto, RSEM, Salmon, STAR, and Stringtie [33–37]. Gene counts obtained for genes on the pseudoautosomal region of the Y chromosome were excluded from further analysis, as they are identical in annotation and counts to these genes on chromosome X. For Kallisto and Salmon, transcript-level expression values were condensed to gene-level values using tximport [38]. Expression matrices for differential expression input were generated using custom scripts as well as the prepDE.py script provided at the Stringtie website. Ten iterations of differential expression analysis were run using different, randomly-chosen combinations of classical and nonclassical monocyte samples, with 3, 4, 5, 6, 7, 8, 9, 12, or 15 samples per group, using the same ten combinations for all workflows and read depths, as shown in Additional file 2. We kept sample combinations consistent for a given sample number when varying read depth to better isolate the effects of decreasing read depth. However, our sample combinations were restricted by the initial read depths of the samples (Additional file 1) and therefore some samples were excluded from individual analyses. Specifically, three samples were excluded from the two highest read depths (classical09, nonclassical01, and nonclassical10), and an additional nine samples were excluded from the highest read depth (classical01, classical04, classical10, classical15, classical16, nonclassical06, nonclassical07, nonclassical13, and nonclassical17). Because of these exclusions, we were unable to test 15 samples per group at the two highest read depths and 12 samples per group at the highest read depth. Differentially expressed genes were identified with Ballgown, DESeq2, edgeR exact test, limma coupled with voom transformation, NOISeqBIO, and SAMseq [39–44]. Of these, all but Ballgown and SAMseq used intrinsic filtering or recommended extrinsic filtering of genes prior to testing. All differential expression tools were specified within the tool commands to run at a detection level of alpha of 0.05 or FDR of 0.05. In general, all software was run with default parameters; specific runtime parameters and software versions are listed in Additional file 3, and scripts for running all code are available at https://github.com/cckim47/kimlab/tree/master/rnaseq. Further information about implementation is available upon request. Note that tool and genome versions have been updated since our previous paper [7], so performance metrics may differ slightly.

### Preparation of reference datasets

Reference datasets were prepared from four published studies of classical and nonclassical monocytes conducted on microarray or BeadChip platforms and retrieved from the NCBI gene expression omnibus (GEO) with accession numbers GSE25913, GSE18565, GSE35457, GSE34515 [27–30], as previously described [7]. A third division of monocytes, intermediate monocytes, has recently been established [45], and these were isolated together with nonclassical monocytes in the two microarray experiments [28, 30], but not in the BeadChip and RNA-Seq data sets [7, 27, 29]. Significant differentially expressed genes between classical and nonclassical monocytes were identified for each dataset with significance analysis of microarrays (SAM) [46] with an FDR of 0.05, and limma [41], with a BH-adjusted *p*-value of 0.05. Performance of the RNA-Seq workflows against both the SAM and limma analyzed microarray data were previously compared to one another and found to exhibit good reproducibility regardless of the statistical method used to analyze the microarray data [7]; as such, we chose to use the genes at the intersection of the two methods for our final reference gene sets here

### Quantification of recall and precision

Both performance metrics were calculated as previously described [7]. Because absolute recall and precision values are influenced by the repertoire of analytes that can be measured by a given platform, following significance testing, we filtered each reference and RNA-Seq gene set to include only features measurable both by RNA-Seq (i.e., present in the GRCh38 genome release, Gencode version 26) and by the microarray (i.e., a probe targeting the feature was present on the microarray platform) within a given comparison. All gene set counts are reported based on these filtered numbers, as are all calculations of recall and precision. Recall was calculated as the number of significant genes in the intersection of the test RNA-Seq dataset with the reference dataset, divided by the number of genes identified as significant in the reference dataset. Precision was calculated as the number of significant genes in the intersection of the test RNA-Seq dataset with the reference dataset, divided by the number of genes identified as significant in the test RNA-Seq dataset.

### Literature survey

To survey current sample number practices in the RNA-Seq literature, the following PubMed search was queried in January 2018: ((rna seq OR rna-seq OR RnaSeq)) AND (differential OR differentially) NOT (miRNA OR non-coding OR “single cell” OR lncRNA OR “circular RNA”). There was no date-based selection, but the earliest studies in the randomly chosen datasets were from 2010. Results were either unfiltered or filtered on species “human”. Only studies which performed differential expression analysis were included. Studies utilizing previously published datasets, including large-scale sequencing efforts (such as TCGA) were excluded, to ensure a representative sampling of the most common experimental designs. For the human-specific survey, only studies utilizing primary patient samples or cell lines derived from individual patients were included. Results of either filtered or unfiltered searches were randomized, and then reviewed sequentially until 100 papers meeting inclusion criteria were reviewed (Additional file 4). Average sample number was determined by adding the number of samples included in each pairwise comparison, divided by two times the total the number of comparisons in a given study. Sample number was corroborated by two authors for 10% of papers with 90% concordance between two reviewers. In studies in which there was discordance in the average sample number calculated, both authors independently reviewed the study and were in agreement following this review.

## Results

### Generation of subsampled real-world RNA-Seq dataset for benchmarking

We sought to empirically assess the impact of read depth and sample number on RNA-Seq workflow performance, using patient-derived clinical samples, which integrate many sources of variability that are not well represented in typical benchmarking datasets. Our RNA-Seq dataset has been previously described [7]. In brief, RNA from nonclassical and classical monocytes was isolated from cryopreserved PBMCs collected as part of a study of Ugandan children. A total of 16 classical and 16 nonclassical patient monocyte samples were utilized in this study. RNA-Seq libraries were sequenced as 51-base single-end reads on an Illumina HiSeq 2500. Total reads per sample were variable, ranging from 6 to 37 million, but with no significant difference of read number or quality between the 16 classical and 16 nonclassical samples [7]. Raw fastq files were randomly subsampled to create fastq files of 3 × 10^4^, 5 × 10^4^, 1 × 10^5^, 3 × 10^5^, 5 × 10^5^, 1 × 10^6^, 2 × 10^6^, 5 × 10^6^, 1 × 10^7^, and 2 × 10^7^ reads for each sample, as allowed for by the original read depth (Additional file 1).

### Overview of empirical testing

As previously described, we used four studies which explored expression differences between classical and nonclassical monocytes, using microarray and BeadChip analysis [27–30], to generate a reference of biological truth for comparison. By utilizing data from four independent studies we were able to minimize the effect that any individual preparation had on the results, while still analyzing clinical samples with inter-sample variability and effect size characteristics commonly found in real-world RNA-Seq studies, but not in traditional benchmarking datasets. Despite differences in collection and processing methods, as well as variability in the genetic backgrounds between studies, in our previous analysis we found that the performance of various RNA-Seq workflows was remarkably consistent when using any of the four reference datasets as truth [7]. As such, we have chosen to report only on performance averaged across the four datasets for the current analysis. Additionally, we previously found strong concordance between results when using either SAM or limma to detect differentially expressed genes from the microarray and BeadChip datasets [7], so have used the intersection of the two analysis methods to generate our “ground-truth” gene lists.

With these four datasets as our references for performance comparisons, we focused our evaluation of RNA-Seq analysis workflows on those which we had previously identified as “high performers” -- high recall, high precision, or among the top in combined performance [7]. From within this subset, we additionally selected for commonly used workflows, ease of implementation, and run speed. Finally, to constrain our exploration space, we limited our analysis to workflows that would ultimately lead to differential expression testing done on read counts (as opposed to FPKM or TPM), and on gene level data (as opposed to transcript level). Based on our previous analysis [7] that found that the differential expression analysis tool had the largest effect on performance, we predicted that it would be more informative to include more differential expression detectors than read aligners or expression modelers. In total, we evaluated four read aligners, five expression modelers, and six differential expression detectors, as shown in Table 1, in thirty total combinations. We applied these workflows to ten iterations of randomly selected samples at each of the following number of samples per group: 3, 4, 5, 6, 7, 8, 9, 12, and 15. Because only 16 samples per condition were available, the higher sample number iterations had more overlapping samples than the lower sample number iterations. The same 10 sample iterations were used at all read depths and across all workflows for consistency among comparisons. If there were not enough samples available for 10 distinct sample combinations at a given read depth, the sample number/read depth combination was not run. To benchmark performance, we calculated the precision (intersecting significant genes divided by total number of significant genes identified by RNA-Seq) and recall (intersecting significant genes divided by the total number of significant reference genes) of each iteration.

**Table 1:** Analysis tools used in this study

Read aligner / Expression modeler	Differential expression tool
HISAT2/Stringtie	Ballgown
Kallisto/Kallisto	DESeq2
Salmon/Salmon	edgeR
STAR/RSEM	limma-voom
STAR/STAR	NOISeqBio
	SAMseq

Additional details are available in Additional file 3

### Differential influence of workflow stages

**Fig. 1:**
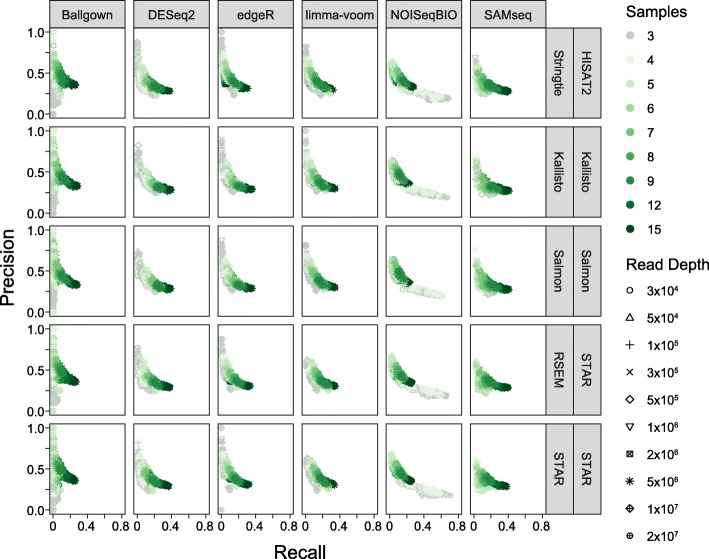
Analysis workflow steps' impact on performance. Precision and recall for each iteration, separated by read aligner and expression modeler (rows) and differential gene tool (columns). Colors represent sample number and shapes represent read depths

For each workflow consisting of all three steps (read alignment, expression modeling and differentially expressed gene identification), we evaluated the ability to detect genes differentially expressed between classical and nonclassical monocytes, at each aforementioned read depth and sample number, over 10 iterations of sample combinations at each sample number. We first wanted to examine the relative impact of each step on precision and recall, over the search space. Strikingly, it was visually clear that the choice of differential expression tool was much more impactful than the choice of the read aligner/expression modeler pair, with performance tending to cluster by differential expression tool over iterations at each read depth and sample number (Fig. 1 and Additional file 5; figures show the same data with shape and color labels inverted). For example, Ballgown showed a large range of precision values across all read alignment and expression modeling pairs whereas NOISeqBio consistently exhibited a large range in recall values. This is similar to our previous finding that the choice of differential expression tool was the most impactful workflow step, in the absence of read or sample subsampling [7]. Given the similarity in performance across different upstream steps when holding differential expression tool constant, we have chosen to subsequently highlight the differential expression tool rather than complete workflow in figures, for ease of comparison between differential expression tools. To facilitate a more in-depth exploration of the data, we also provide an interactive figure that enables visualization of performance metrics for individual workflows (Additional file 6).

We note that we have compared significant gene lists to each of four microarray datasets individually and then calculated an average performance across the datasets. Since any two of the truth datasets exhibited at least 500 unique differentially detected significant genes in a direct comparison [7], it is not surprising that absolute precision was not high when calculated with each truth dataset, and supports the hypothesis that there would be variability across independently collected and analyzed patient monocyte samples. It is likely that this and other factors play a combinatorial role in explaining the low absolute precision values and demonstrate the difficulty of defining a ground truth for genome-wide studies. As such, we advise focusing on the relative comparisons of various workflows’ precision/recall trade-off which provides useful guidance when weighing options for RNA-Seq analysis.

### Effects of read depth on performance

**Fig. 2:**
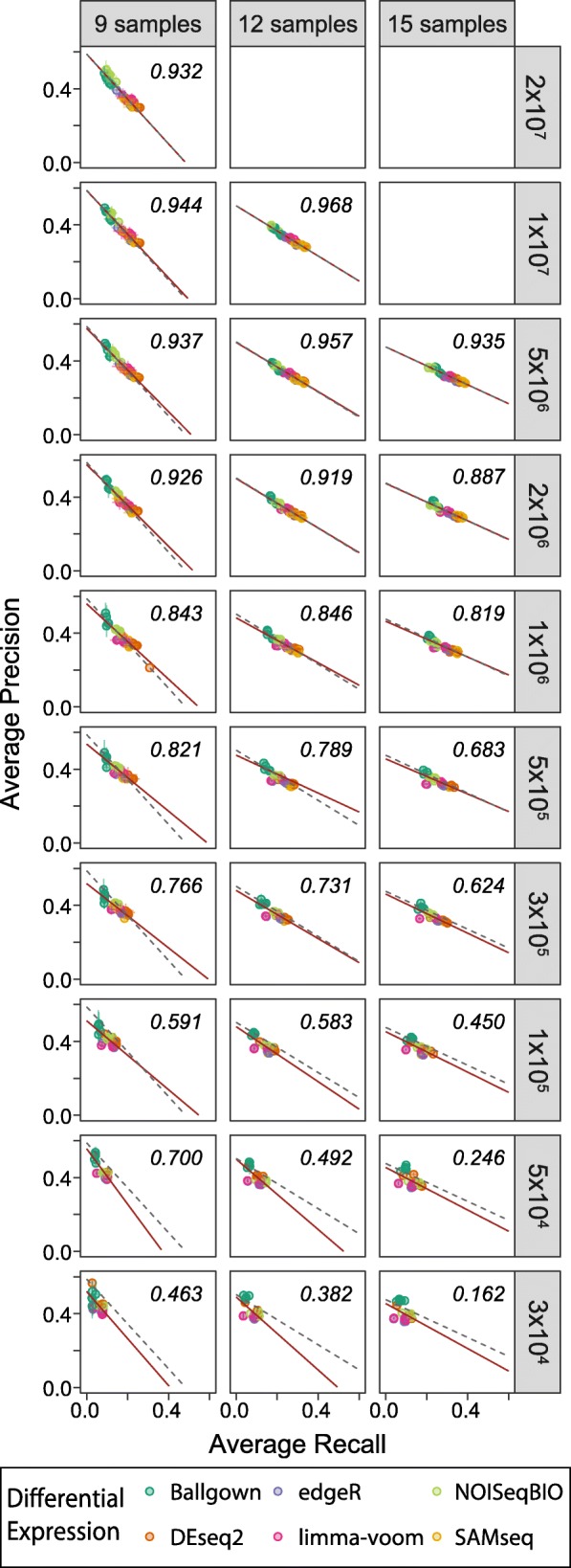
Read depth's impact on performance. Precision and recall, averaged over the 10 iterations at a given sample number and read depth, split by sample number (columns) and read depth (rows). Values for each workflow (read aligner, expression modeler, and differential expression tool) are averaged and displayed separately. Points represent mean; bars represent standard deviation; colors represent differential expression tool. Red solid line represents linear regression line for plotted data. R^2^ value corresponds to plotted data. Gray dashed line represents linear regression fit of the first row of data for each column, superimposed over subsequent rows for comparison

Within each workflow, performance varied dramatically as read depth and sample number per group varied. To isolate the effect of read depth on the precision and recall of the workflows, we focused on iterations run on the highest numbers of samples per group to minimize the impact of sample number effect on interpretation. At the higher read depths, a performance trade-off between precision and recall was present when comparing workflows, following an inverse linear relationship (Fig. 2), as we previously reported [7]. To aid in visual comparison of performance at each read depth to the original, highest read depth performance, we have depicted the original regression line at each subsequent sub-sampled read depth. This inverse linear relationship degrades as read depth decreases, primarily due to a loss in recall. Initial degradation in linearity becomes apparent at 2 × 10^6^ reads, with a drop in correlation, and the majority of workflows deviate from the high-read regression line by 1 × 10^5^ reads (Fig. 2 and Additional file 7). This was seen consistently at sample numbers of 15, 12 and 9, with only slight variation in the pattern of degradation at the different sample numbers. Throughout the range of read depths, differential expression tools largely maintain their precision and recall positions relative to other tools (Additional file 7 and Additional file 8), although Ballgown’s recall with nine samples more quickly degraded than the other tools as read depth decreased. Limma-voom and edgeR coupled with HISAT2-Stringtie also lost recall more rapidly than when coupled with the other read aligner / expression modeler combinations, at all three sample numbers (Fig. 2 and Additional file 6). At these higher sample numbers, NOISeqBIO was comparatively resilient to effects of decreasing read depth, with maintenance of its balance between precision and recall across the tested depths (Additional file 6). Of note, SAMseq was unable to consistently handle the lower read depths, with the majority of iterations failing at 1 × 10^5^ reads and all iterations failing at 3 × 10^4^ read depth (Additional file 9). SAMseq’s performance improved as read depth increased, and was able to run with no failures at the two highest read depths (Additional file 9). While the inner workings of any individual tool are beyond the scope of this study, it appears that the failures were due to errors following SAMseq’s read depth estimation, when the function samr.estimate.depth() returns an estimated read depth of 0 for at least one sample in each failed comparison. Given that initial estimates for RNA-Seq read depth requirements were estimated to be one to two orders of magnitude higher than the failure depths [12, 13], it is likely SAMseq was not developed to handle these lower read depths. Of note, increasing sample size does decrease the number of failures from SAMseq for the intermediate-high read depths (Additional file 9), suggesting an interaction between these parameters.

### Effects of sample number on performance

**Fig. 3:**
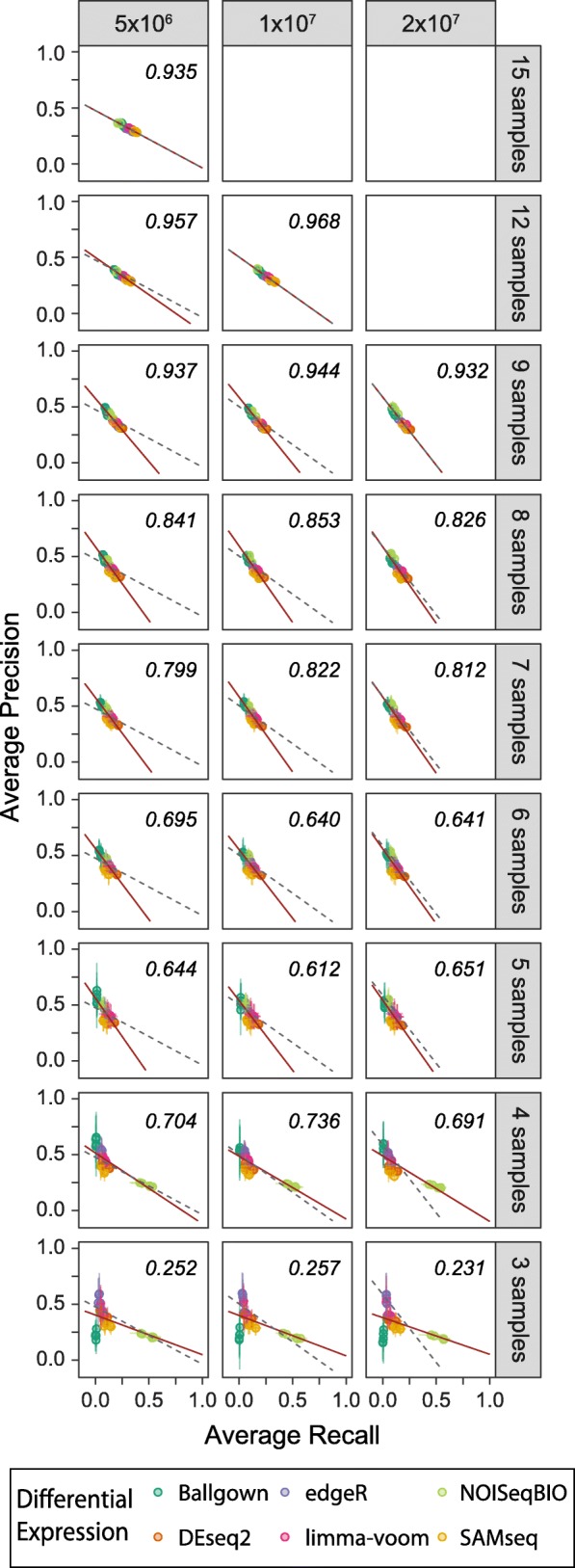
Sample number's impact on performance. Precision and recall, averaged over the 10 iterations at a given sample number and read depth, split by read depth (columns) and sample number (rows). Values for each workflow (read aligner, expression modeler, and differential expression tool) are averaged and displayed separately. Points represent mean; bars represent standard deviation; colors represent differential expression tool. Red solid line represents linear regression line for plotted data. R^2^ value corresponds to plotted data. Gray dashed line represents linear regression fit of the first row of data for each column, superimposed over subsequent rows for comparison

Decreasing read depths consistently led to decreased performance—particularly decreased recall—at high sample numbers; however, it was clear that the slope of the recall-precision relationship shifted as sample number changed (Fig. 2). To more closely examine the effects of sample number, we limited examination to the highest read depths. Surprisingly, reduction in sample number was impactful from a relatively high number of samples. Changes in the slope of the recall-precision relationship were apparent from eight samples per group, with a large decrease in the recall-precision linearity relationship at six samples per group (Fig. 3). Similar to our findings with read depth, this change was most reflected by loss of recall, disproportionate to the loss of precision (Fig. 3 and Additional file 7). Notably, Ballgown performed particularly poorly at the lowest sample numbers, with many iterations failing to call any significant genes, leading to precision and recall values of zero (Fig. 3). This poor performance was not related to the upstream choice of read aligner and expression modeler, but rather the same sample groupings tended to return poor results for all upstream workflow combinations. As noted by the Ballgown authors, although Ballgown shares a similar underlying linear model to limma for identification of differentially expressed genes, the initial empirical Bayes modeling employed by limma prior to differential testing allows for shrinkage of variance estimates, which has a larger effect for smaller sample sizes where less biological replicate information is available [44]; thus, limma has superior performance at low sample numbers, as we see here. NOISeqBIO also demonstrated unusual behavior at the lowest sample numbers – at three and four samples per group, performance skewed heavily towards recall, with very low precision, the opposite of the performance seen at higher sample numbers. This behavior was independent of read depth (Fig. 3 and Additional file 7). NOISeqBIO combines a non-parametric model with an empirical Bayes approach to shrink variance estimates, regardless of sample number. However, when NOISeqBIO is used with fewer than five samples per group, there is a change in the methodology for the creation of the null distribution of its non-parametric model. At these lower sample numbers, k-means clustering is employed to identify genes with similar expression patterns and information is shared between these genes when creating the null distribution, whereas this is not done at higher sample numbers [42]. This difference in methodology for managing lower sample numbers might explain the abrupt shift to high recall with reduced precision.

**Fig. 4:**
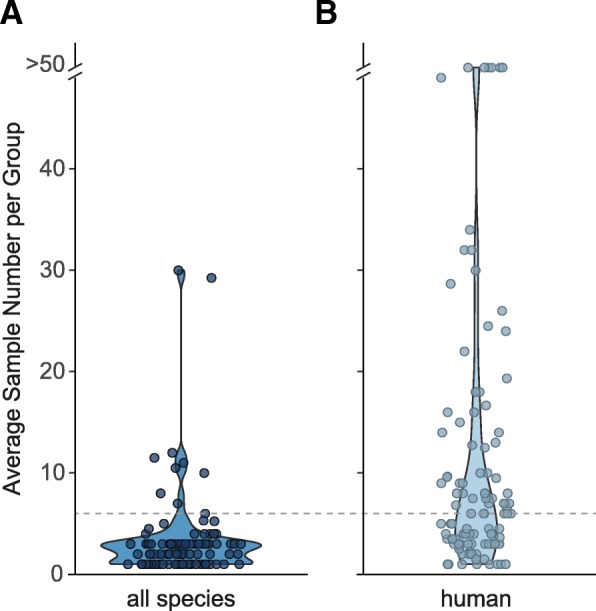
Literature survey of RNA-Seq experiment sample numbers. Violin plots of sample numbers used in 200 studies containing RNA-Seq differential gene expression analysis, either from all species (**a**) or limited to primary human samples (**b**). Individual dots represent average sample number used in each study. Grey dashed line represents six samples

Given the somewhat surprising result that sample numbers below six had severely reduced performance, we next sought to assess how widely used low sample numbers were in recent RNA-Seq studies. From 100 randomly chosen studies, over 90% used six or fewer samples per group (Fig. 4a). When this survey was repeated and restricted to studies of human samples, the average sample numbers were slightly higher, with about half of the studies falling at or below six samples per group (Fig. 4b). This suggests that while some authors of human studies recognize the increased variability inherit to clinical samples and increase sample size accordingly, the performance characteristics of many human studies would be improved with increased sample numbers. Given these results, caution should be exercised in interpreting many recent RNA-Seq studies that may conform to common experimental design approaches, but that may be underpowered for RNA-Seq analysis. Additionally, this highlights the necessity of benchmarking RNA-Seq tool performance using datasets most similar to those that the methods will be applied to, to better define best practices for study design and analysis.

### Correlations with significant gene numbers

**Fig. 5:**
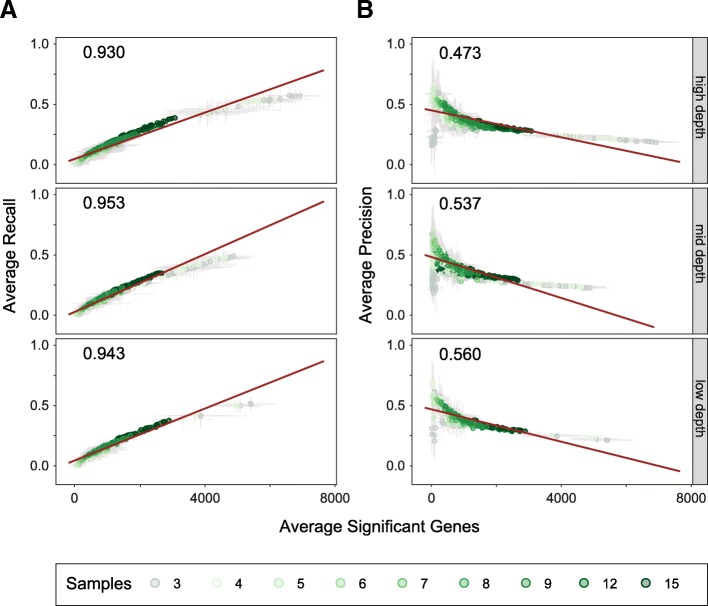
Significant gene number's impact on performance. Average recall (**a**) or average precision (**b**) versus the average number of genes identified as significant. Panels are split by read depths, with 2 × 10^7^, 1 × 10^7^, 5 × 10^6^, and 2 × 10^6^ reads plotted as high read depths, 1 × 10^6^, 5 × 10^5^ and 3 × 10^5^ plotted as medium read depths, and 1 × 10^5^, 5 × 10^5^, and 3 × 10^4^ plotted as low read depths. Dots represent values for individual workflows (read aligner, expression modeler, and differential expression tool) at a given sample number and read depth, averaged over the ten sample combination iterations run at each given sample number and read depth. Bars represent standard deviation. Colors represent sample number. Red line represents linear regression for plotted data. R^2^ value corresponds to plotted data

In our initial study examining the performance of workflows, we observed that the number of genes called significant by a workflow heavily influenced the recall and precision, with a strong correlation between recall and the number of genes identified as significant, and an inverse relationship between precision and the number of significant genes [7]. As such, we hypothesized that changes in the number of genes identified as significant would be correlated with the degradation of performance at lower sample numbers and read depths. As predicted, we observe a strong relationship between recall and number of genes called significant, with the number of genes called significant tending to increase as sample number increased, with a commensurate increase in recall (Fig. 5a and Additional file 10). Surprisingly, and in contrast to our previous observations across workflows [7], the converse was not true for precision (Fig. 5b). While the trend that higher numbers of genes called significant tended to have lower precision remained true, this effect was much less pronounced. Interestingly, the precision across workflows tended to decrease at the highest sample numbers. While this could represent an increase in falsely called genes as the total number of significant genes increases, it is also possible that at these higher sample numbers RNA-Seq overtakes microarray’s and BeadChip’s abilities to detect differentially expressed genes. Notably, workflows employing NOISeqBIO at three and four samples called the highest number of significant genes of any workflows, which likely accounts for the relatively high recall with poor precision displayed by these workflows at low sample numbers. This suggests that results from NOISeqBIO must be interpreted with caution at low sample numbers, due to the higher likelihood of type I error.

## Conclusions

Of the workflows examined, all performed well at higher read depths and sample numbers, and the choice of workflows at these parameters should be largely influenced by the tolerance of a specific application for type I versus type II error, as we concluded previously [7]. However, caution should be used at lower read depths and sample numbers, as performance is variable and highly dependent on the choice of differential expression tool, with much smaller impact from read aligner and expression modeler. These results also give insight into the read depth and sample number required for robust results when designing RNA-Seq experiments involving clinical samples, which exhibit more genetic and pre-analytical heterogeneity than typical in vitro study designs. Performance was relatively resistant to changes in read depth, with very minimal impact down to two million reads, which is considerably lower than previously published suggested read depths and may reflect analysis of different organisms and/or sample types [10–13]. Conversely, tool performance—particularly recall and the commensurate number of genes called significant—rapidly declined as sample number per group decreased, with changes apparent even by eight samples. At six or fewer samples per group, tool choice became increasingly impactful with SAMseq and Ballgown falling below the linear relationship, and thus being “worse” performers in this context. These findings corroborate past suggestions that increasing biological replicates will generally have a greater impact than increasing read depth [8, 10, 14, 15], although this also depends on the “starting points” for read depth and sample number per group. If sample number is constrained, caution must be exercised in choosing a differential expression tool, as performance is more variable. Specifically, there is increased risk of Type II error, most disproportionately when using Ballgown with the lowest sample numbers, and Type I error in the case of NOISeqBIO used with fewer than five samples. These findings represent a departure from current practices used in many studies, which tend to follow more traditional experimental designs employing fewer replicates.

## Additional files

Additional file 1: Original read depth of individual samples. (XLSX 12 kb)

Additional file 2: Sample combinations for each iteration at varying sample numbers. The same sample combinations were run at all read depths and for all workflows. (XLSX 21 kb)

Additional file 3: Table of software tools, with versions and runtime parameters. (XLSX 15 kb)

Additional file 4: Literature survey citations and average sample number. 200 studies containing RNA-Seq differential expression analysis, either from all species or limited to primary human samples. Average sample number from these studies is also displayed in Fig. 4. (XLSX 52 kb)

Additional file 5: Analysis workflow steps' impact on performance. Precision and recall for each iteration, separated by read aligner and expression estimator (rows) and differential gene tool (columns). Colors represent read depths and shapes represent sample number. These are the same data presented in Fig. 1 with color and shape labels switched. (PDF 2300 kb)

Additional file 6: Interactive figure for comparison of performance metrics. (A) Absolute precision and recall for each workflow. (B) Relative ranks of precision and recall for each workflow. Grey dots show performance for all workflows for the selected read depth(s) and sample number(s); red dots highlight the selected workflow(s). (XLSX 536 kb)

Additional file 7: Impact on performance by read depth and sample number. Precision and recall, averaged over the 10 iterations at a given sample number and read depth, split by sample number (columns) and read depth (rows). Values for each workflow (read aligner, expression modeler, and differential expression tool) are averaged and displayed separately. Points represent mean; bars represent standard deviation; colors represent differential expression tool. Red line represents Lm fit for plotted data. Text is the corresponding R^2^ value. (PDF 9556 kb)

Additional file 8: Impact on rank performance by read depth and sample number. Rank precision and rank recall, averaged over the 10 iterations at a given sample number and read depth, split by sample number (columns) and read depth (rows). Values for each workflow (read aligner, expression modeler, and differential expression tool) are averaged and displayed separately. Points represent mean; bars represent standard deviation; colors represent differential expression tool. Red line represents Lm fit for plotted data. Text is the corresponding R^2^ value. (PDF 9675 kb)

Additional file 9: Number of SAMseq failed iterations. Iteration was counted as a failure if SAMseq was not successfully run due to an error message. Bars represent count of failures, colored by read depth. (PDF 142 kb)

Additional file 10: Number of significant genes by number of biological replicates. Bar represents average number of significant genes for a given read depth, sample number, and differential expression tool. Average was calculated by averaging each of the ten sample combination iterations at a given sample number and read depth, for all five read aligner/expression modeler combinations upstream of a given differential expression tool. Standard deviation is shown. Colored by read depth. (PDF 786 kb)

## Abbreviations

FDR: False discovery rate
FPKM: Fragments per kilobase of transcript per million mapped reads
PBMCs: Peripheral blood mononuclear cells
RNA-Seq: RNA sequencing
TPM: Transcripts per million
