# Supplementary figures and images for: Empirical assessment of the impact of sample number and read depth on RNA-Seq analysis workflow performance
Source: BMC Bioinformatics. 2018 Nov 14;19:423. doi: 10.1186/s12859-018-2445-2 (PMC6234607; doi:10.1186/s12859-018-2445-2)

Precision

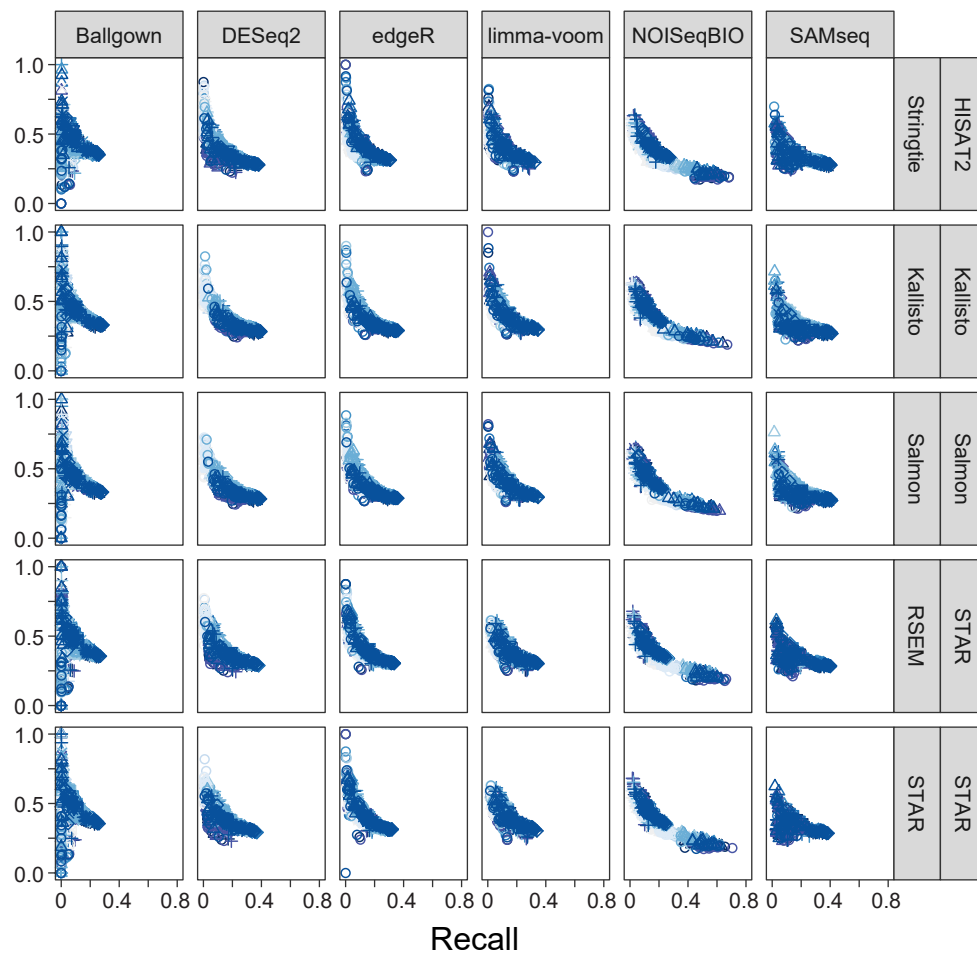

Samples

- 3
- △ 4
- + 5
- × 6
- ◇ 7
- ▽ 8
- ⊠ 9
- \* 12
- ⊕ 15

Read Depth

- $3 \times 10^4$
- $5 \times 10^4$
- $1 \times 10^5$
- $3 \times 10^5$
- $5 \times 10^5$
- $1 \times 10^6$
- $2 \times 10^6$
- $5 \times 10^6$
- $1 \times 10^7$
- $2 \times 10^7$

Supplement: Supplementary file 5 — Analysis workflow steps' impact on performance. Precision and recall for each iteration, separated by read aligner and expression estimator (rows) and differential gene tool (columns). Colors represent read depths and shapes represent sample number. These are the same data presented in Fig. 1 with color and shape labels switched. (PDF 2300 kb) [file 12859_2018_2445_MOESM5_ESM.pdf]

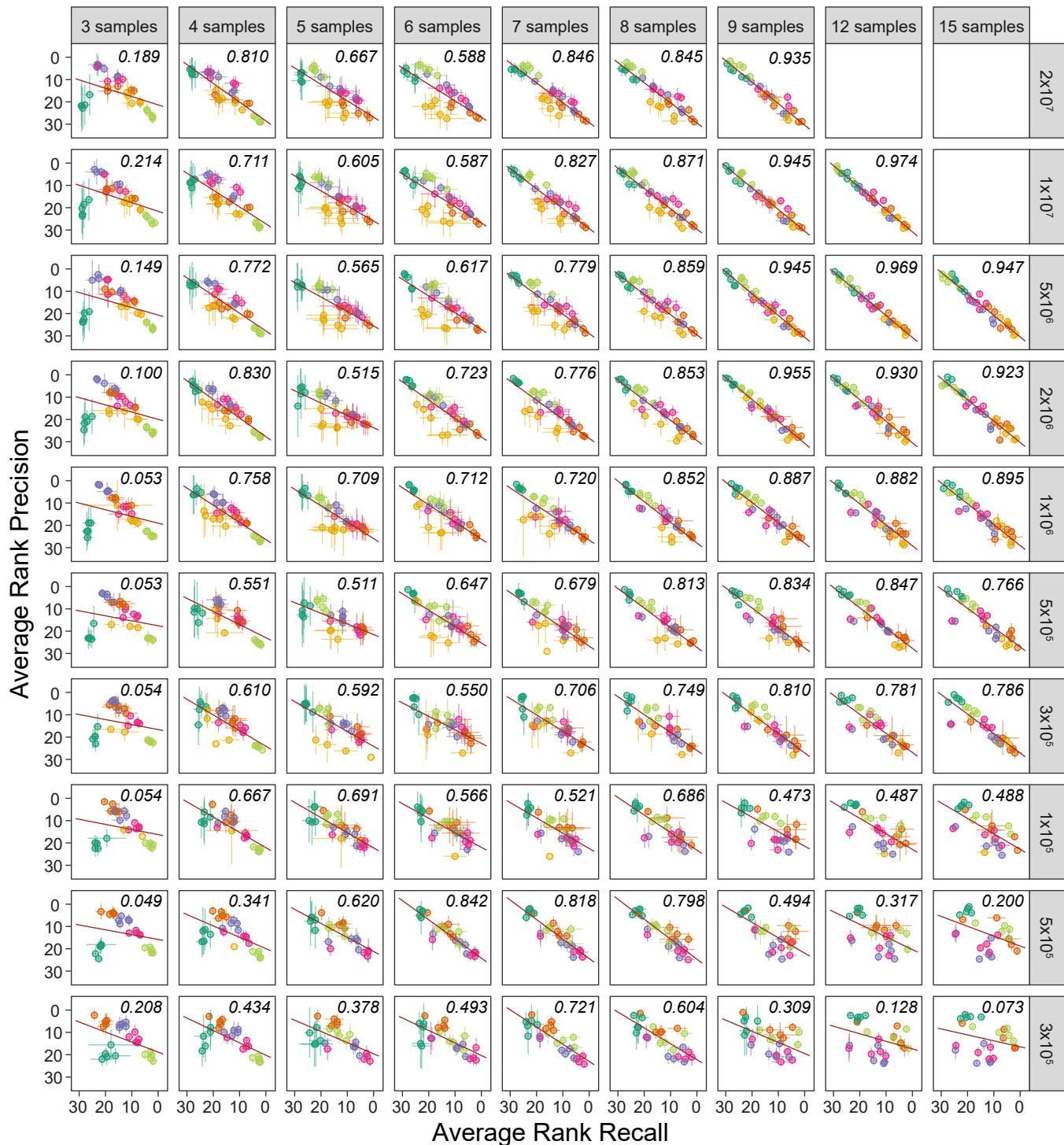

Differential  
Expression

Ballgown

edgeR

NOISeqBIO

DESeq2

limma-voom

SAMseq

Supplement: Supplementary file 8 — Impact on rank performance by read depth and sample number. Rank precision and rank recall, averaged over the 10 iterations at a given sample number and read depth, split by sample number (columns) and read depth (rows). Values for each workflow (read aligner, expression modeler, and differential expression tool) are averaged and displayed separately. Points represent mean; bars represent standard deviation; colors represent differential expression tool. Red line represents Lm fit for plotted data. Text is the corresponding R2 value. (PDF 9675 kb) [file 12859_2018_2445_MOESM8_ESM.pdf]

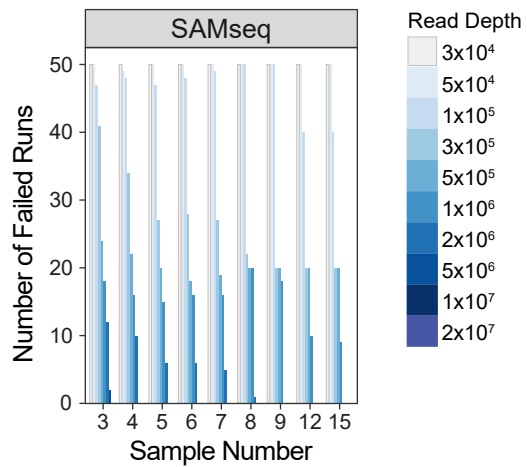

Supplement: Supplementary file 9 — Number of SAMseq failed iterations. Iteration was counted as a failure if SAMseq was not successfully run due to an error message. Bars represent count of failures, colored by read depth. (PDF 142 kb) [file 12859_2018_2445_MOESM9_ESM.pdf]

Average Significant Genes

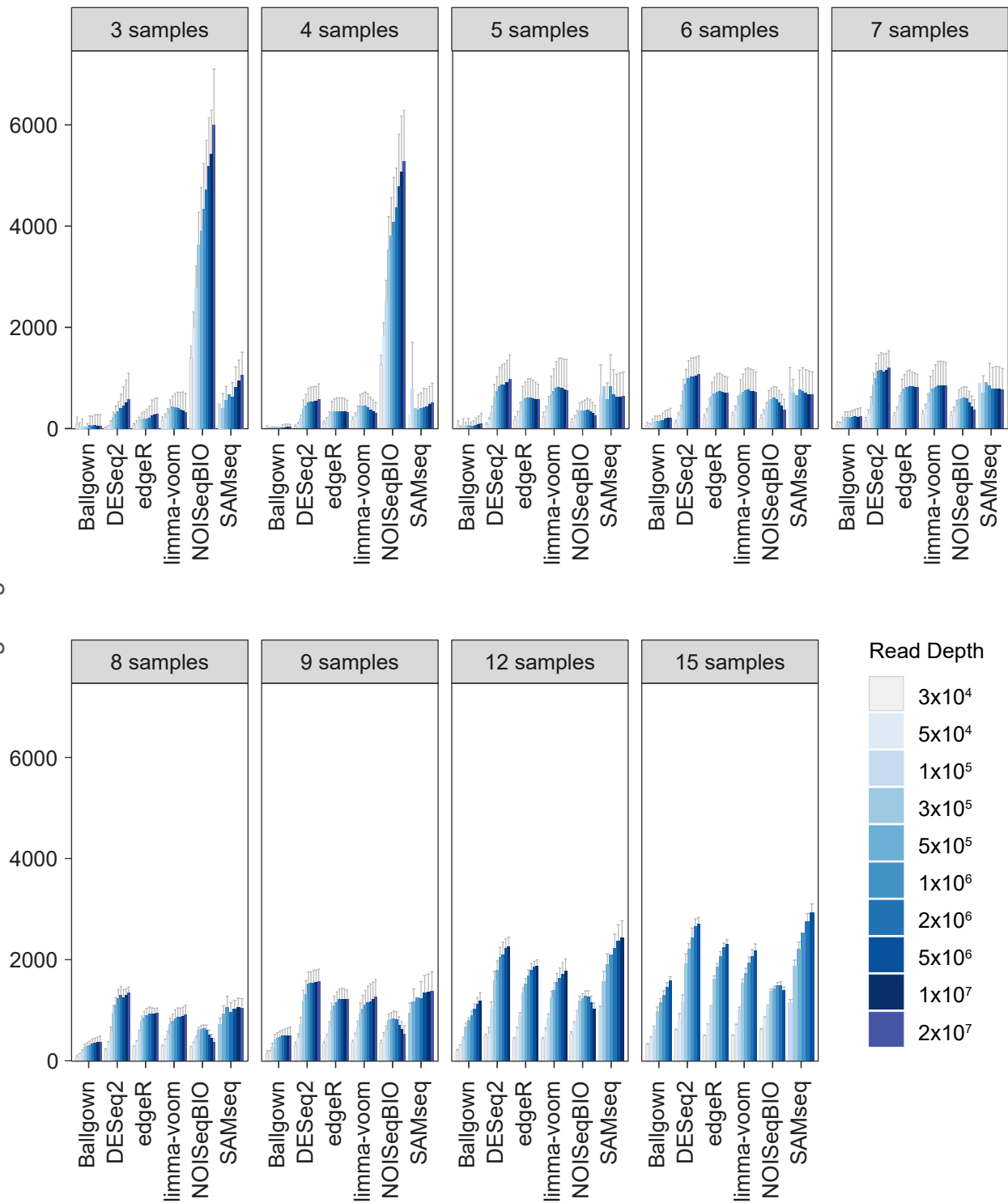

Supplement: Supplementary file 10 — Number of significant genes by number of biological replicates. Bar represents average number of significant genes for a given read depth, sample number, and differential expression tool. Average was calculated by averaging each of the ten sample combination iterations at a given sample number and read depth, for all five read aligner/expression modeler combinations upstream of a given differential expression tool. Standard deviation is shown. Colored by read depth. (PDF 786 kb) [file 12859_2018_2445_MOESM10_ESM.pdf]
