# Supplementary material for: Empirical assessment of the impact of sample number and read depth on RNA-Seq analysis workflow performance
Source: BMC Bioinformatics. 2018 Nov 14;19:423. doi: 10.1186/s12859-018-2445-2 (PMC6234607; doi:10.1186/s12859-018-2445-2)
Supplement: Supplementary file 7 — Impact on performance by read depth and sample number. Precision and recall, averaged over the 10 iterations at a given sample number and read depth, split by sample number (columns) and read depth (rows). Values for each workflow (read aligner, expression modeler, and differential expression tool) are averaged and displayed separately. Points represent mean; bars represent standard deviation; colors represent differential expression tool. Red line represents Lm fit for plotted data. Text is the corresponding R2 value. (PDF 9556 kb) [file 12859_2018_2445_MOESM7_ESM.pdf]

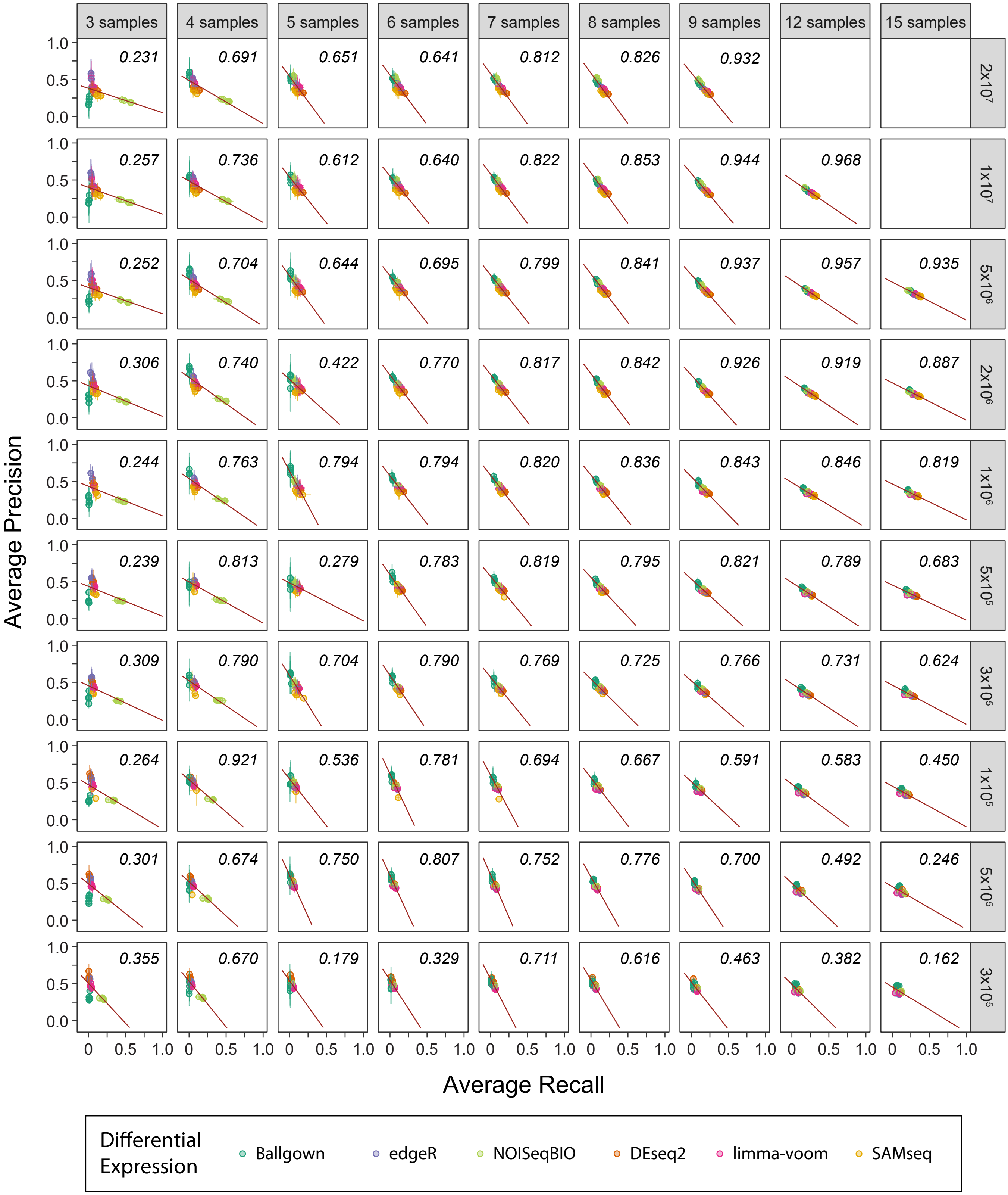

3 samples
4 samples
5 samples
6 samples
7 samples
8 samples
9 samples
12 samples
15 samples
$2x10^7$
0.231
0.691
0.651
0.641
0.812
0.826
0.932
$1x10^7$
0.257
0.736
0.612
0.640
0.822
0.853
0.944
0.968
$5x10^6$
0.252
0.704
0.644
0.695
0.799
0.841
0.937
0.957
0.935
$2x10^6$
0.306
0.740
0.422
0.770
0.817
0.842
0.926
0.919
0.887
$1x10^6$
0.244
0.763
0.794
0.794
0.820
0.836
0.843
0.846
0.819
$5x10^5$
0.239
0.813
0.279
0.783
0.819
0.795
0.821
0.789
0.683
$3x10^5$
0.309
0.790
0.704
0.790
0.769
0.725
0.766
0.731
0.624
$1x10^5$
0.264
0.921
0.536
0.781
0.694
0.667
0.591
0.583
0.450
$5x10^5$
0.301
0.674
0.750
0.807
0.752
0.776
0.700
0.492
0.246
$3x10^5$
0.355
0.670
0.179
0.329
0.711
0.616
0.463
0.382
0.162
Average Precision
Average Recall
0.0
0.5
1.0
Differential Expression
Ballgown
edgeR
NOISeqBIO
DEseq2
limma-voom
SAMseq
